# Stress-dependent ribosome-associated proteomics reveals a role for Sis1 in thermotolerance and virulence in *Cryptococcus neoformans*

**DOI:** 10.1128/spectrum.00104-26

**Published:** 2026-09-21

**Authors:** Maia F. Mazzaferro, Alexandra C. Benedetto, Amanda L. M. Bloom, Prabhakar Singh, Shichen Shen, Jun Qu, John C. Panepinto

**Affiliations:** 1 Department of Microbiology and Immunology, Witebsky Center for Microbial Pathogenesis and Immunology, Jacobs School of Medicine and Biomedical Sciences, University at Buffalo, SUNY 192705 , Buffalo, New York, USA; 2 Department of Pharmaceutical Sciences, School of Pharmacy and Pharmaceutical Sciences, University at Buffalo, SUNY 15497 , Buffalo, New York, USA; University of Florida College of Dentistry, Gainesville, Florida, USA

**Keywords:** ribosome remodeling, *Cryptococcus*, stress-responsive translation, Sis1, heat shock proteins

## Abstract

During human infection, *Cryptococcus neoformans* can disseminate to the central nervous system, causing often-fatal cryptococcal meningitis in individuals with compromised immunity. To acutely adapt to host micro-environments, *C. neoformans* employs mechanisms to reprogram its translatome to provide cells with tools to survive the stressors of the human host. The identities and functions of ribosome-associated factors that may modulate this process in *C. neoformans* remain unknown. We hypothesized that following host-related stressors, specific proteins associate with ribosomes and influence stress adaptation in *C. neoformans*. Utilizing a proteomic approach, termed RiboPROT, we analyzed the protein composition of mRNA-associated ribosomes from cells grown under unstressed (30°C), thermally stressed (shifted to 37°C), and oxidatively stressed (shifted to 2 mM H_2_O_2_) conditions. We identified unique factors enriched with actively translating ribosomes during host-related stressors. Phenotypic analysis of deletion mutants in selected ribosome-associated factors revealed that the J-domain protein Sis1 is important for growth at elevated temperature, Hsp70 and Hsp90 function, and virulence. The *sis1*Δ mutant also exhibited sensitivity to multiple drugs that cause mitochondrial perturbation, and growth on acetate as a sole carbon source suppressed the thermal growth defect at 38°C, suggesting that Sis1 supports mitochondrial homeostasis during thermal stress. Additionally, the *sis1*Δ mutant displayed transient hyper-repression of translation initiation during a shift to acute thermal stress at human body temperature but not at human febrile temperature. Global features of stress-responsive translatome reprogramming remained intact in the absence of Sis1, suggesting its role in thermotolerance and virulence is likely linked to regulation of specific targets involved in mitochondrial homeostasis in *C. neoformans*.

**IMPORTANCE:**

Fungal pathogens are an underrepresented and emerging global public health threat. In 2022, the World Health Organization recognized *Cryptococcus neoformans* as the top fungal high-priority pathogen, signifying the urgency for increased research and new therapeutics. The mechanisms by which *C. neoformans* adapts to the insults of the human body and causes systemic disease remain incompletely understood. Although the ribosome is essential to fungal viability and is targetable by small molecules, the conservation of the eukaryotic ribosome hampers the development of molecules that are selective for the fungal ribosome. Here, we identify stress-dependent ribosome-associated factors in *C. neoformans* and highlight one identified protein, Sis1, demonstrating its contribution to thermotolerance and virulence. Identification of stress-induced ribosome-associated factors and uncovering their biological role in the cell may identify pathways for selective therapeutic targeting of the fungal ribosome.

## INTRODUCTION

Despite the estimated ~5 million fungal species, only ~200 species are identified to be human-associated as pathogens or commensals (1). Even fewer cause invasive systemic infections by overcoming the collaboration of mammalian endothermy and host immune defenses that provide protection from invasive fungal infections (2). The World Health Organization named 19 invasive fungal diseases of top priority to signify increased research emphasis and the need for novel therapeutics; *Cryptococcus neoformans* ranked at the top of the list (3, 4). Ubiquitously found within the environment, *C. neoformans* is an encapsulated basidiomycete with key virulence traits that allow for survival within a human host (5). A defining key virulence trait is thermotolerance, which confers the ability to adapt to mammalian core body temperature of 37°C and febrile temperatures of 38°C–42°C (6). The geography of cryptococcal disease incidence overlaps with significant barriers to treatment due to a lack of necessary healthcare infrastructure and resources. Current front-line antifungal therapeutics used to combat cryptococcal meningitis are toxic, difficult to access, and have been hampered by the development of resistance (7), highlighting the need for novel drug targets and the development of safe, effective, and low-cost anticryptococcal therapies.

The decoding of genetic information stored in mRNAs into functional proteins via translation is an essential process for all cells and is carried out by ribosomes. Inhibition of translation remains a particularly strong target for antimicrobial drug development in prokaryotic organisms (8). However, shared eukaryotic ancestry presents challenges to the targeting of fungal ribosomes while limiting human host toxicity (9). Thus, targeting the core process of translation through ribosome inhibition has limited promise as a therapeutic target for the development of antifungal drugs (10). With these challenges, more selective antifungal targets, such as targeting proteins that modulate ribosomal activity, may provide a better drug target than the ribosome itself.

To establish infection and survive the many insults of the human body, *C. neoformans* adapts by reprogramming its translatome to allow the translation of stress-responsive effectors (11). In response to stressors, such as thermal stress or reactive oxygen species (ROS) from immune cells, levels of homeostatic pro-growth mRNAs, such as those encoding the abundant ribosomal protein (RP) transcripts, are repressed (11, 12). This leads to the reallocation of translational machinery for the prioritization of stress-responsive transcripts that promote adaptation. We have identified and documented key functional steps in this process, including that the removal of pro-growth mRNAs from the translating pool occurs via Ccr4-mediated deadenylation and that regulation of translation initiation occurs through phosphorylation of the α subunit of eIF2 via the activation of Gcn2, the sole eIF2α kinase of *C. neoformans* (6, 13, 14).

Historically, ribosomes were thought to play a passive role in translation regulation, acting as homogeneous machines. More recently, the framework has shifted to a deeper understanding of ribosomes as both sensors of cellular status and platforms for the recruitment of regulatory factors in other organisms (15–17). The specific stress-dependent ribosome interactors and their impact on stress adaptation and pathogenesis in fungal pathogens such as *C. neoformans* remain unexplored.

We hypothesized that following host-related stressors, specific proteins associate with ribosomes and influence stress adaptation in *C. neoformans*. To test this, we utilized a proteomic approach, RiboPROT, to assess stress-specific ribosome-associated proteins using a combination of polysome profiling and mass spectrometry. Here, we report the identification of unique factors enriched with actively translating ribosomes in *C. neoformans* following exposure to host-related stress conditions. Of the identified factors, we selected three for further investigation due to links to translation in other systems or the absence of human orthologs: the heat shock chaperone proteins (Hsps) Hsp104 (CNAG_07347), J-domain protein Sis1 (CNAG_06106), and a hypothetical protein (CNAG_05167). Of these, only a *sis1*Δ mutant exhibited a defect in thermal stress adaptation. Because thermotolerance is a key virulence trait in *C. neoformans*, Sis1 was chosen for further mechanistic investigation.

Sis1 is a Hsp40/J-domain protein co-chaperone that cooperates with Hsp70s to aid in maintaining protein homeostasis (18). In the model yeast *Saccharomyces cerevisiae*, *SIS1* is essential, associates with the 40S ribosomal subunit, and is required for translation initiation (19, 20). Investigation of the mechanism underlying this thermal stress sensitivity revealed sensitivity to mitochondrial perturbation, as well as sensitivity to inhibitors of Hsp70 and Hsp90. Furthermore, the *sis1*Δ mutant exhibited a defect in virulence in the *Galleria mellonella* model of cryptococcosis at 38°C. This work validates that stress-specific ribosome-associated proteins can impact stress adaptation and pathogenesis in *C. neoformans*. Further work will explore additional ribosome-associated proteins identified from this study and identify additional ribosome-associated proteins under other host-relevant conditions.

## RESULTS

### RiboPROT reveals ribosome-associated proteins during acute host-like stressors in *C. neoformans*

To identify stress-dependent interactors of the ribosome, we utilized a peptide-centric bottom-up proteomics approach termed RiboPROT. This method integrated polysome profiling and mass spectrometry to identify unique protein associations at the ribosome during human-like stress conditions (Fig. 1A). Wild-type (WT) cells (H99) were grown to mid-log phase at 30°C (unstressed; NOTREAT), shifted to 37°C for 60 min (TEMP), or treated with 2 mM hydrogen peroxide for 60 min (ROS). Polysomes were fractionated, collected, and pooled for further proteomic analysis. To specifically identify ribosome-associated proteins from proteins that co-sediment in the gradients, we ran parallel samples treated with the chelating agent, ethylenediaminetetraacetic acid (EDTA). EDTA chelates magnesium ions required for the binding of the 40S and 60S ribosomal subunits, leading to their dissociation (21). Proteomic analysis of the EDTA-treated samples was then used to exclude co-sedimented proteins and thus increase confidence in identifying ribosome-associated factors (File S1). Data are available via ProteomeXchange with identifier PXD082145.

**Fig 1:**
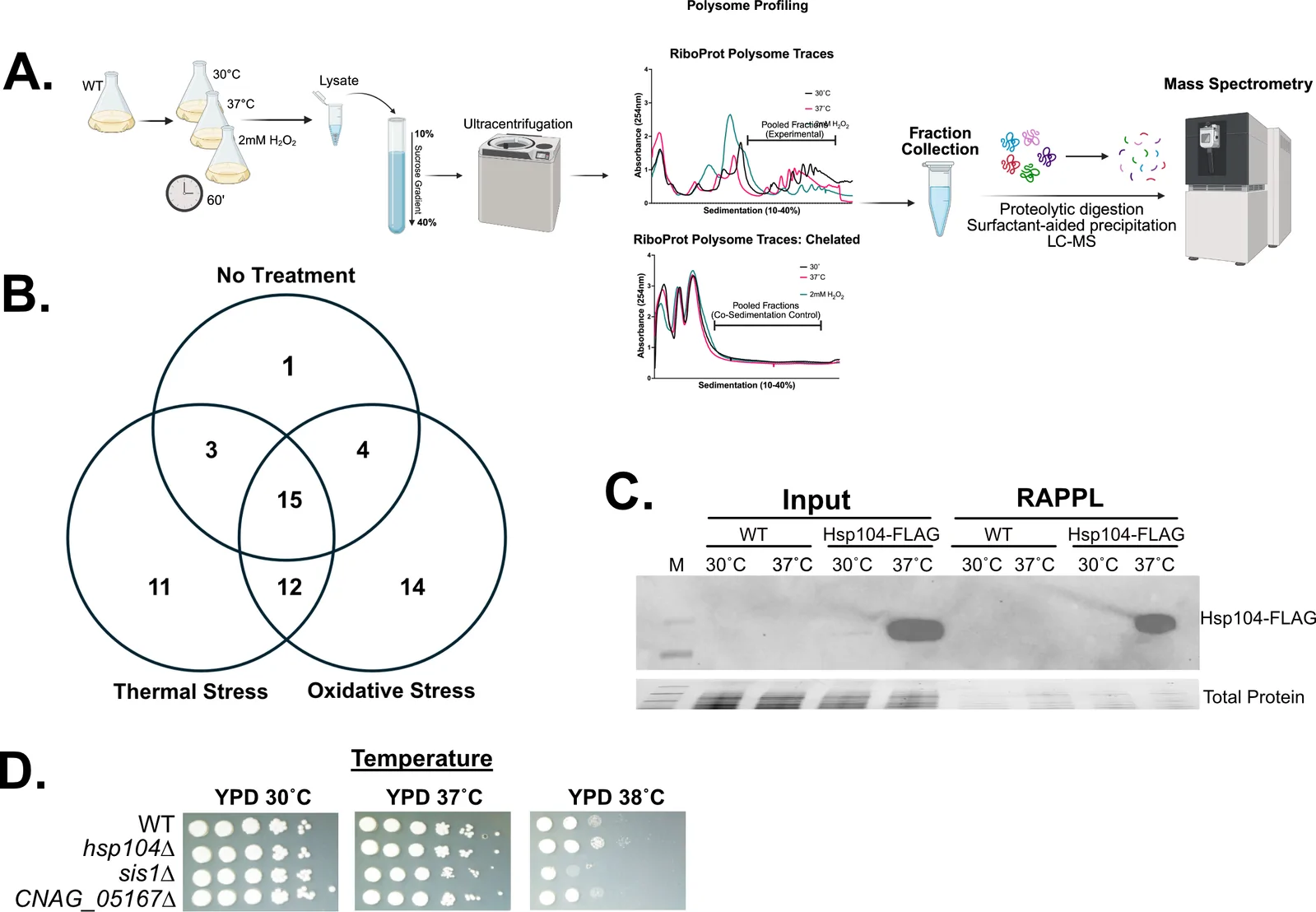
RiboPROT reveals ribosome-associated proteins during acute host-like stressors in *C. neoformans*. (**A**) Experimental design of the RiboPROT workflow. WT cells were grown to mid-logarithmic phase in 30°C YNB + 2% dextrose media, then shifted to 30°C YNB + 2% dextrose (control), 37°C YNB + 2% dextrose (temperature), or YNB + 2% dextrose + 2 mM H_2_O_2_ (ROS) for 60 min. Cultures were prepared for polysome profiling and split for an EDTA-treated control. Peptides were separated by nano-reversed-phase liquid chromatography (LC) and detected by mass spectrometry (MS). Peptide sequences were identified by *in silico* analysis (*n* = 4). Schematic created with BioRender. (**B**) Venn diagram indicating the overlap in ribosome-associated proteins between the control (30°C) and experimental (37°C and H_2_O_2_) conditions that were present in at least three of four replicates and not found in the EDTA control groups. (**C**) Representative western blot of WT and Hsp104-FLAG total cell lysate (input) and ribosome affinity purification (RNA affinity purification using poly-lysine [RAPPL]) from cultures grown to mid-log phase at 30°C or shifted to 37°C for 1 h (*n* = 3). (**D**) Indicated strains were grown overnight in YPD media at 30°C, washed, and resuspended in sterile water to an OD_600_ of 1.0. Ten-fold serial dilutions were spotted on YPD agar plates and incubated for 3 days at the indicated temperature. Plates were photographed following 72 h of growth. Shown is one representative biological replicate (*n* = 3).

Gene ontology enrichment analysis demonstrated that genes related to translation, structural constituents of the ribosome, and ribosomes were significantly enriched, confirming the isolation of fractions containing ribosomes (File S2; Table S1). To prioritize and define ribosome-associated protein hits for each condition, we identified and filtered proteins that were absent from the EDTA-treated conditions and found in at least three out of the four biological replicates of our experimental conditions. Through this, we found ribosome-associated protein hits shared among conditions as well as hits specific to each condition. We identified 11 unique thermal stress-dependent associations and chose to further explore the unique thermal stress-dependent associations (Fig. 1B; File S3).

To validate our RiboPROT approach, we utilized RNA affinity purification using poly-lysine (RAPPL) and western blotting to assess the ribosome association of Hsp104 under thermal stress conditions. We performed RAPPL on a FLAG-tagged Hsp104 strain from cultures grown to mid-log phase at 30°C or shifted to 37°C for 60 min, replicating the culturing conditions for the initial RiboPROT experiment (Fig. 1C). No signal was seen in our WT control, confirming specificity. Hsp104-FLAG was strongly detected in our RAPPL eluates following 60 min of 37°C thermal stress and minimally detected in our untreated input, consistent with our RiboPROT result that identified Hsp104 as associating with ribosomes under thermal stress. Although we attempted to complement our *sis1*Δ mutant with a tagged allele, neither a C-terminal nor N-terminal FLAG tag supported functional complementation (File S4). Fortunately, experimental evidence from other systems supports the ribosome association of Sis1 (19, 20, 22).

We next investigated selected candidate hits identified by RiboPROT, with a focus on ribosome-associated proteins under thermal stress (File S3; Table S2). We selected chaperones Hsp104 (CNAG_07347) and J-domain protein Sis1 (CNAG_06106), both of which have roles in translation in *S. cerevisiae*, and a hypothetical protein (CNAG_05167), an uncharacterized yet highly expressed gene with no human ortholog. Many of the remaining temperature-specific hits were metabolic enzymes and not pursued further because their deletion would likely lead to confounding phenotypes unrelated to ribosome association. To ensure that our selected hits were associating with the ribosome and not just nascent polypeptides, we mapped the peptides across the entire protein (File S5). We concluded that our selected protein hits did not have an N-terminus bias, thus supporting their role as a ribosome-associated protein rather than a nascent polypeptide.

We generated *hsp104*Δ, *sis1*Δ, and *CNAG_05167*Δ mutants by standard methods in the H99 background strain and performed serial dilution spot plate analyses to assess growth at human body temperature (37°C) and human febrile temperature (38°C) (Fig. 1D). Whereas all three mutants grew like the wild type at 37°C, the *sis1*∆ mutant exhibited thermosensitivity at 38°C. Given the clear role for Sis1 in thermal adaptation in *C. neoformans,* we selected the *sis1*Δ mutant for further study.

### Sis1 is required for optimal growth and virulence at human febrile temperature

Given that the *sis1*Δ mutant exhibited a thermal stress phenotype at human febrile temperature in our initial screen, we transformed the *sis1*Δ mutant with a construct expressing wild-type *SIS1*. Expression of *SIS1* in the *sis1*Δ background restored wild-type growth at 38°C, indicating the thermosensitivity of the *sis1*Δ mutant was due to the intended mutation (Fig. 2A). Because Sis1 is a chaperone regulator, we next asked if *SIS1* gene expression was responsive to thermal stress. We assessed *SIS1* expression in wild-type *C. neoformans* following a shift from 30°C to 37°C using northern blot probing for *SIS1* mRNA. We found that *SIS1* mRNA levels increase during thermal stress (Fig. 2B), consistent with a role as a heat shock protein.

**Fig 2:**
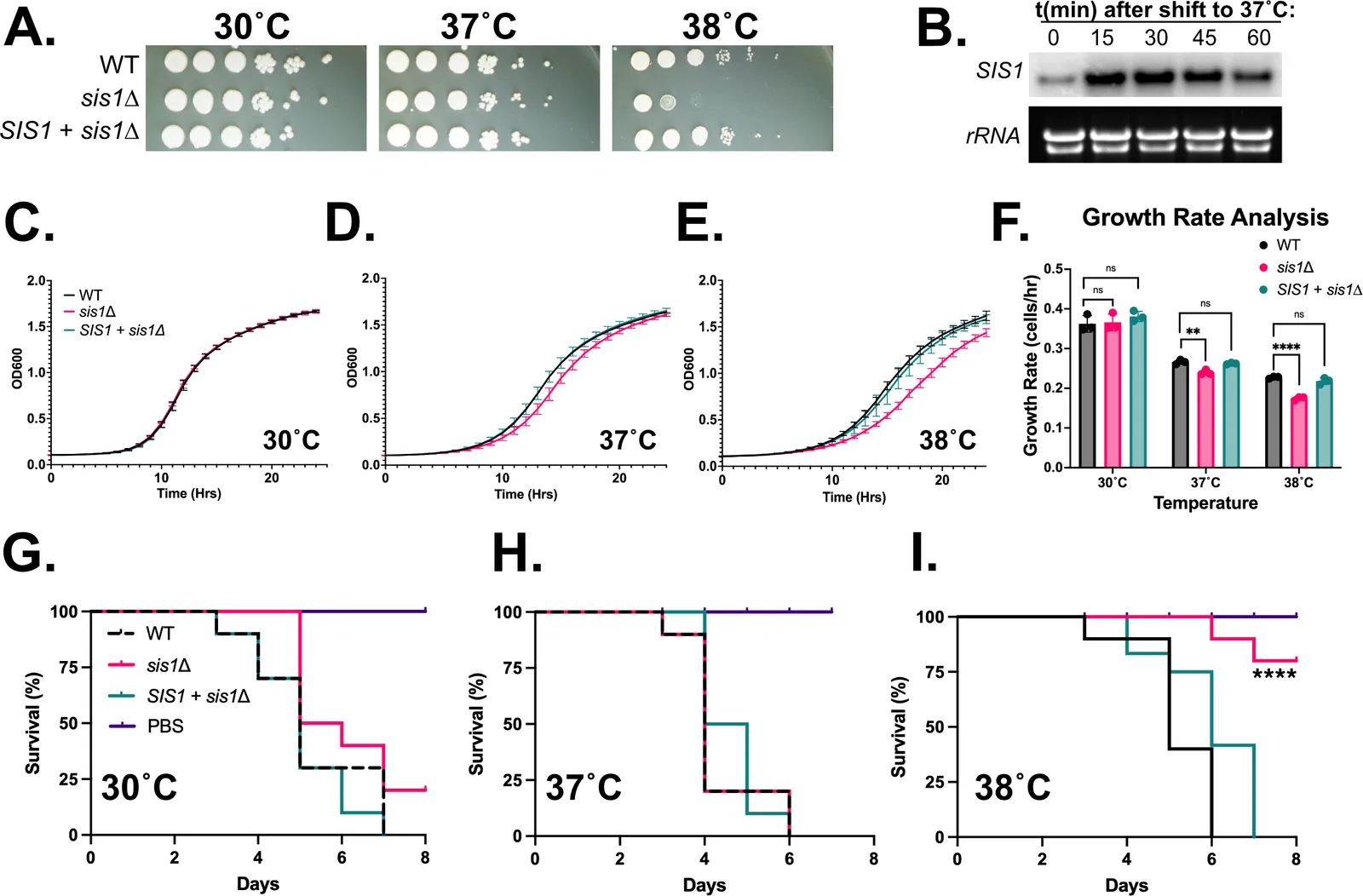
Sis1 is required for optimal growth and virulence at human febrile temperature. (**A**) Serial dilution spot plate analysis of WT, *sis1*∆, and *SIS + sis1*∆ on YPD agar grown at the indicated temperature. Shown is one representative biological replicate (*n* = 3). (**B**) Northern blot analysis of *SIS1* mRNA abundance in WT in a time course following a shift from 30°C to 37°C for 60 min. One representative biological replicate is shown (*n* = 3). (**C–E**) Growth curve analysis of WT, *sis1*∆, and *SIS + sis1*∆. Cultures were seeded from overnight cultures at an OD_600_ of 0.10 in 100 µL of YPD media and grown for 24 h at the indicated temperatures. An OD_600_ reading was taken every 15 min. Each curve represents the average of three biological replicates. Error bars represent the standard deviation across three biological replicates (*n* = 3). (**F**) Bar graph indicating the growth rate calculated from the linear phase of growth. Error bars represent the standard deviation across three biological replicates. Significance was calculated by a one-way ANOVA with Dunnett’s *post hoc* test (**, *P* = 0.0015; ****, *P* < 0.0001). (**G–I**) Mortality curves from *Galleria mellonella* larvae infected with WT, *sis1*∆, or *SIS + sis1*∆ and incubated at either 30°C, 37°C, or 38°C. Differences in survival between groups were assessed using the log-rank (Mantel–Cox) test (****, *P* < 0.0001). Shown is one representative replicate (*n* = 3).

To further investigate the role of Sis1 in thermotolerance, we evaluated kinetic growth by growth curve analysis at environmental (30°C), human body temperature (37°C), or human febrile temperature (38°C). At 30°C, we observed no difference in growth between WT and the *sis1*∆ mutant (Fig. 2C); however, by increasing the temperature to 37°C, we observed a small growth defect in the *sis1*∆ mutant (Fig. 2D). This phenotype was more pronounced at 38°C (Fig. 2E). We quantified the growth rate during exponential growth for each temperature condition and found a significant difference between WT and the *sis1*∆ mutant at both 37°C and 38°C, which was restored by the complement (Fig. 2F).

To assess a potential role for Sis1 thermotolerance in connection with *C. neoformans* pathogenesis, we used the *Galleria mellonella* (wax-worm larvae) model (23–25) to investigate virulence (Fig. 2G through I). We found that at human febrile temperature, the loss of Sis1 led to attenuated virulence (Fig. 2I). Together, our data demonstrate that Sis1 contributes to thermotolerance and is required for optimum growth and virulence at human febrile temperature.

### The deletion of Sis1 leads to an altered translational landscape following a shift to human body temperature

Given the identification of Sis1 as a ribosome-associated protein, we next assessed the role of Sis1 in supporting stress-responsive translation in *C. neoformans*. We first assessed the sensitivity of the wild-type, *sis1*Δ mutant, and complemented strain against a panel of translation inhibitors via spot plate assay. We observed that the *sis1*∆ mutant shows a mild sensitivity to higher concentrations of cycloheximide (CHX) at both 30°C and 37°C (Fig. 3A). CHX blocks translation elongation via binding to the E site of the 60S ribosomal subunit, which leads to the inhibition of eEF2-mediated translocation (26). We also observed that the *sis1*∆ mutant exhibited a temperature-dependent sensitivity to 3-amino-1,2,4-triazole (3-AT), an inhibitor of histidine biosynthesis that leads to histidine deficiency and increases uncharged histidyl-tRNAs (Fig. 3A). 3-AT impacts translation by inducing ribosome stalling at histidine codons, resulting in ribosome collisions that activate Gcn2 (27, 28). Activation of Gcn2 downregulates translation initiation as a result of phosphorylation of the translation initiation factor eIF2α (11).

**Fig 3:**
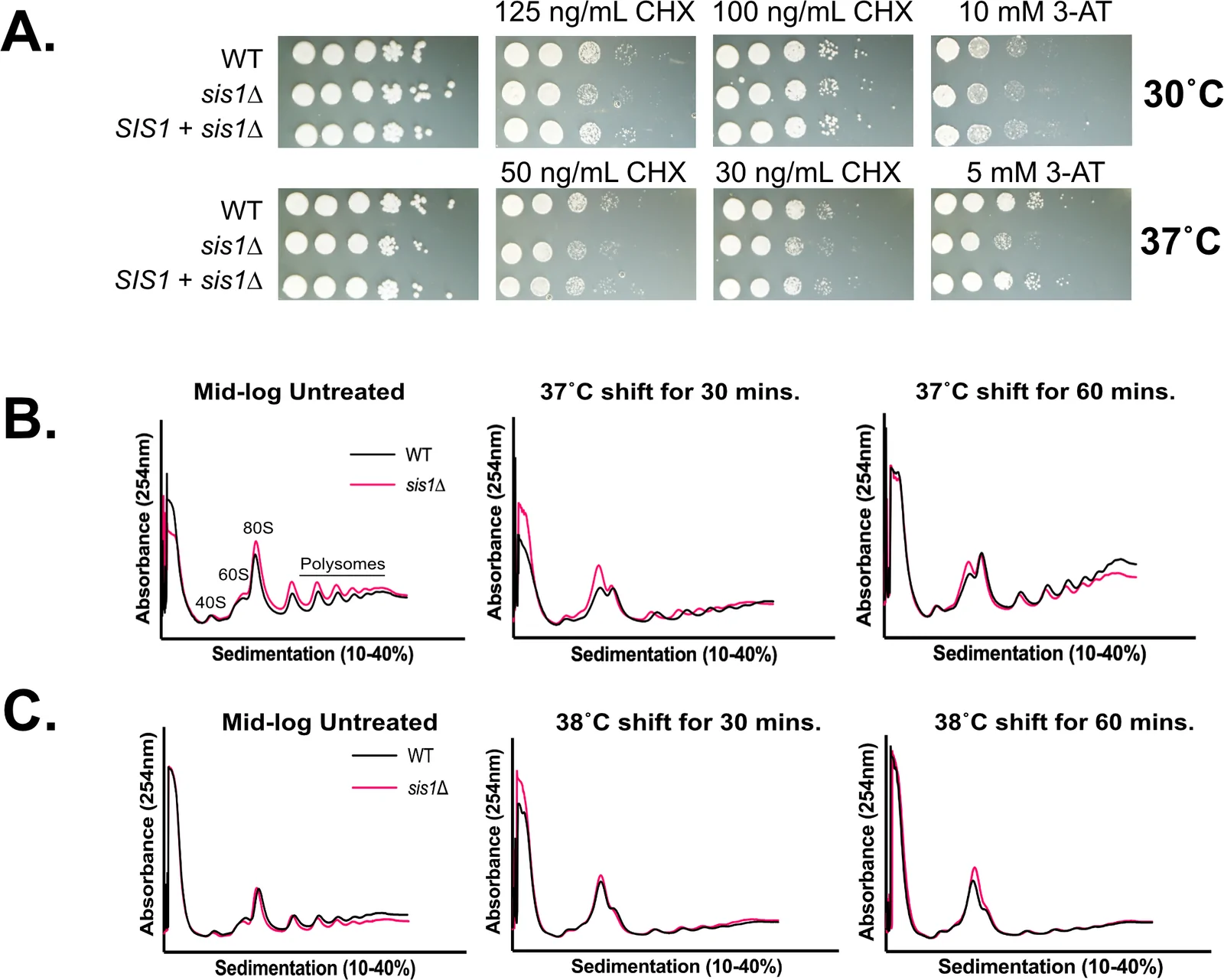
The deletion of Sis1 leads to an altered translational landscape following a shift to human body temperature. (**A**) Spot dilution plate analysis of WT, *sis1*∆, and *SIS + sis1*∆ on YPD agar with the indicated drugs and concentrations, grown at the indicated temperature. Shown is one representative biological replicate (*n* = 3). (**B**) Representative polysome traces comparing the translational state of the wild type or *sis1*∆ mutant grown to mid-log at 30°C or shifted to 37°C for 30 or 60 min. One representative biological replicate is shown (*n* = 3). (**C**) Representative polysome traces comparing the translational state of the wild type or *sis1*∆ mutant grown to mid-log at 30°C or shifted to 38°C for 30 or 60 min. One representative biological replicate is shown (*n* = 3).

Next, we compared the global state of translation following a shift to 37°C in the wild type and *sis1*Δ mutant via polysome profiling (Fig. 3B). In the absence of stress, the polysome profiles of the wild type and *sis1*Δ mutant were indistinguishable, exhibiting robust polysome peaks, a strong 80S and minimal 60S peak, indicating a high level of translation initiation. Following a 30-min shift from 30°C to 37°C, the *sis1*∆ mutant exhibited an increase in the 60S subunit peak relative to the wild type. Interestingly, the 60S peak eclipsed the monosome peak, suggesting a greater repression of translation initiation in the *sis1*Δ mutant relative to the wild type. Both the wild type and *sis1*Δ mutant displayed a decrease in polysomes at 30 min, consistent with reduced initiation. At 60 min post-shift, both the WT and *sis1*∆ mutant recover, as evidenced by an increase in the polysome portion of the trace, suggesting that this difference in translation initiation is transient (Fig. 3B).

Because we observed thermosensitivity following growth at 38°C in our *sis1*∆ mutant, we also investigated the translational landscape following a shift to 38°C via polysome profiling (Fig. 3C). The shift to 38°C resulted in a 60S peak that eclipsed the monosome peak in both the wild type and *sis1*Δ mutant, but both strains appeared similar. After 60 min, the translational repression persisted in both strains. These data suggest that although there are global changes in translational state in response to thermal stress, deletion of *SIS1* has minimal impact on global translation in *C. neoformans*.

### Canonical features of translatome reprogramming are intact in the absence of Sis1 following a shift to human body and febrile temperatures

Our previous work has established a model of stress-responsive translatome reprogramming that involves the removal of pro-growth mRNAs, such as RP transcripts, and the concomitant prioritization of stress-responsive mRNAs (11, 12). To determine if Sis1 contributes to this process during thermal stress, we assessed levels of the *SSA2* mRNA, encoding a thermal stress-inducible Hsp70 family member (29), and *RPL2,* a representative RP transcript that is transiently repressed in response to the 37°C thermal stress (Fig. 4A). Total RNA isolated from the wild type or *sis1*Δ mutant in a time course following a shift from 30°C to 37°C was harvested and fractionated by formaldehyde agarose gel electrophoresis for northern blot and probed simultaneously for *SSA2* and *RPL2*. We found similar induction of *SSA2* expression and *RPL2* repression between WT and the *sis1*∆ mutant (Fig. 4A), suggesting that both the heat shock response and mRNA decay responses are intact in the absence of Sis1. Together, these results suggest that core features of translatome reprogramming are intact in the absence of Sis1.

**Fig 4:**
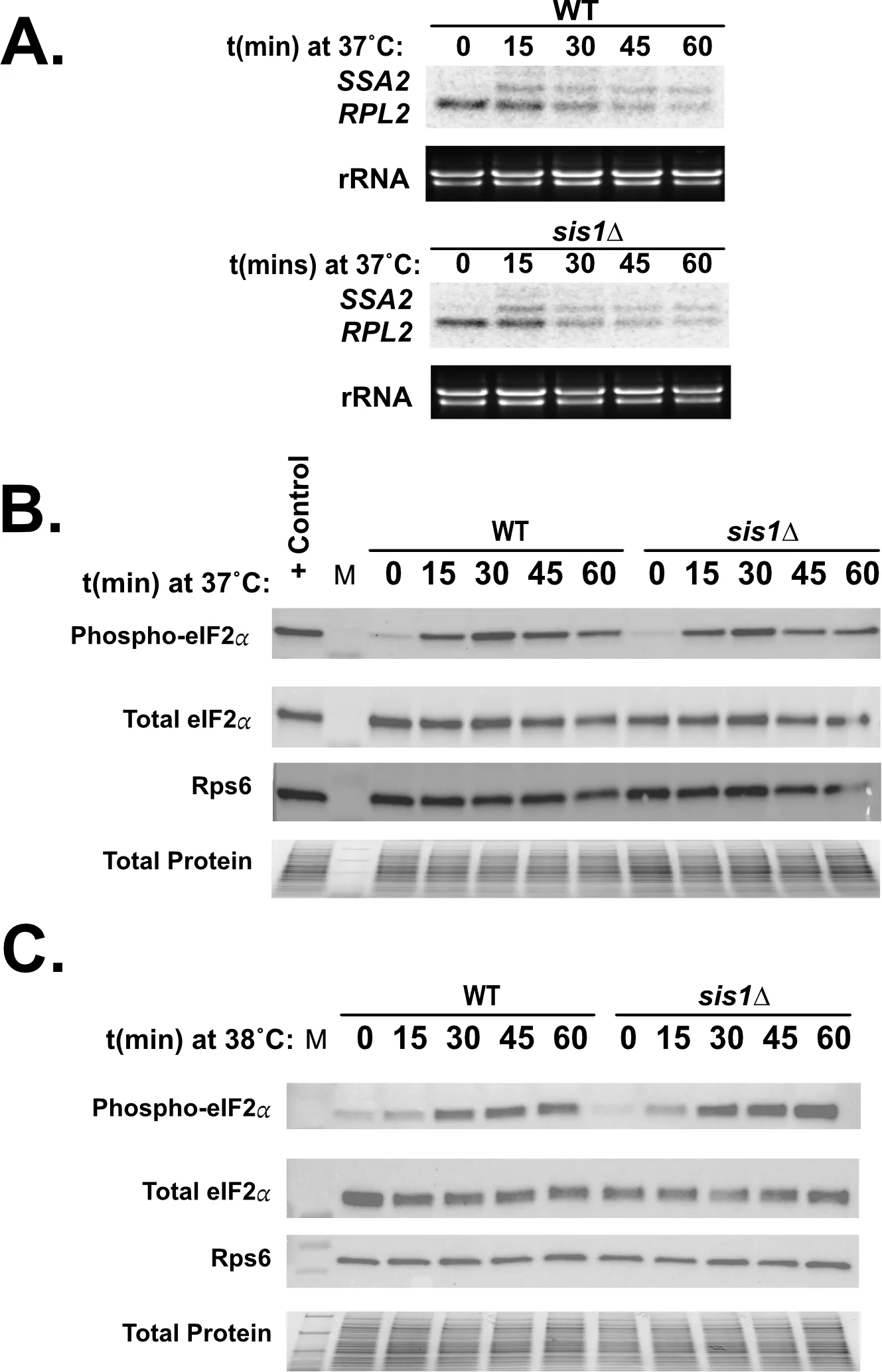
Canonical features of translatome reprogramming are intact in the absence of Sis1 following a shift to human body and febrile temperatures. (**A**) Northern blot analysis of *RPL2* and *SSA2* mRNA abundance in a time course following a shift from 30°C to 37°C for 60 min in WT and the *sis1*Δ mutant. One representative biological replicate is shown (*n = 3*). (**B**) Western blot analysis of phosphorylated eIF2α, total eIF2α, and Rps6 abundance in a time course following a shift from 30°C to 37°C for 60 min in WT and the *sis1*Δ mutant. Total protein serves as a loading control. +, positive control shown is a 2 mM hydrogen peroxide WT sample. One representative biological replicate is shown (*n = 3*). (**C**) Western blot analysis of phosphorylated eIF2α, total eIF2α, and Rps6 abundance in a time course following a shift from 30°C to 38°C for 60 min in WT and the *sis1*Δ mutant. Total protein serves as a loading control. One representative biological replicate is shown (*n = 3*).

Given that we observed sensitivity to 3-AT and increased 60S peak height in response to thermal stress in the absence of Sis1, we next evaluated the activation of Gcn2, the sole eIF2α kinase in *C. neoformans* in the wild type and *sis1*Δ mutant. We used western blotting to observe eIF2α total levels, eIF2α phosphorylation, and Rps6 levels as readouts for Gcn2 activation and total translational capacity. We found no differences in phospho-eIF2α levels between WT and the *sis1*∆ mutant following a thermal shift to 37°C despite the increase in 60S peak height in the polysome profiles (Fig. 4B). This suggests that the increase in 60S peak heights observed in the *sis1*∆ mutant polysomes after 30 min at 37°C may be independent of Gcn2 activation.

Next, we assessed total eIF2α to provide a baseline for phosphorylated levels of eIF2α (Fig. 4B) and observed no difference between WT and the *sis1*∆ mutant. In addition, we assessed Rps6 (Fig. 4B), a component of the 40S ribosomal subunit and regulator of ribosome biogenesis in yeast (30). We observed no differences in Rps6 levels between WT and the *sis1*∆ mutant, suggesting ribosome biogenesis is unaffected by the loss of Sis1. Together, these results confirm that the acute responses of translatome reprogramming are intact in the absence of Sis1 following a shift to human body temperature, 37°C.

Due to the robust thermosensitivity of the *sis1*∆ mutant at human febrile temperatures, we also wanted to assess Gcn2 activation following a shift to the febrile temperature, 38°C, in the absence of Sis1. Although we found no difference in levels of phosphorylated eIF2α, total eIF2α, and Rps6 levels in the *sis1*∆ mutant following a shift to 38°C, we observed a consistent increase in eIF2α phosphorylation across the time course in both wild type and the *sis1*Δ mutant relative to the pattern observed at 37°C, suggesting that the shift to 38°C places additional stress on the translational machinery in *C. neoformans* (Fig. 4C).

### Hsp70/90 dysfunction and mitochondrial defects underlie the *sis1*Δ thermosensitivity

The functional role of Sis1 as a co-chaperone is to promote ATP hydrolysis in Hsp70 proteins and facilitate handoff of Hsp70 clients to the Hsp90 system (31–33). To investigate if deletion of *SIS1* impacts Hsp70 or Hsp90 function, we assessed the *sis1*Δ mutant for sensitivity to inhibitors of Hsp70 (Pifithrin-µ) (34) and Hsp90 (Radicicol) (35, 36). At 30°C, neither inhibitor impacted growth of the *sis1*Δ mutant. At 37°C, however, the *sis1*Δ mutant exhibited sensitivity to both inhibitors (Fig. 5A). This suggests that in the absence of *sis1*Δ, the Hsp70-Hsp90 system is compromised.

**Fig 5:**
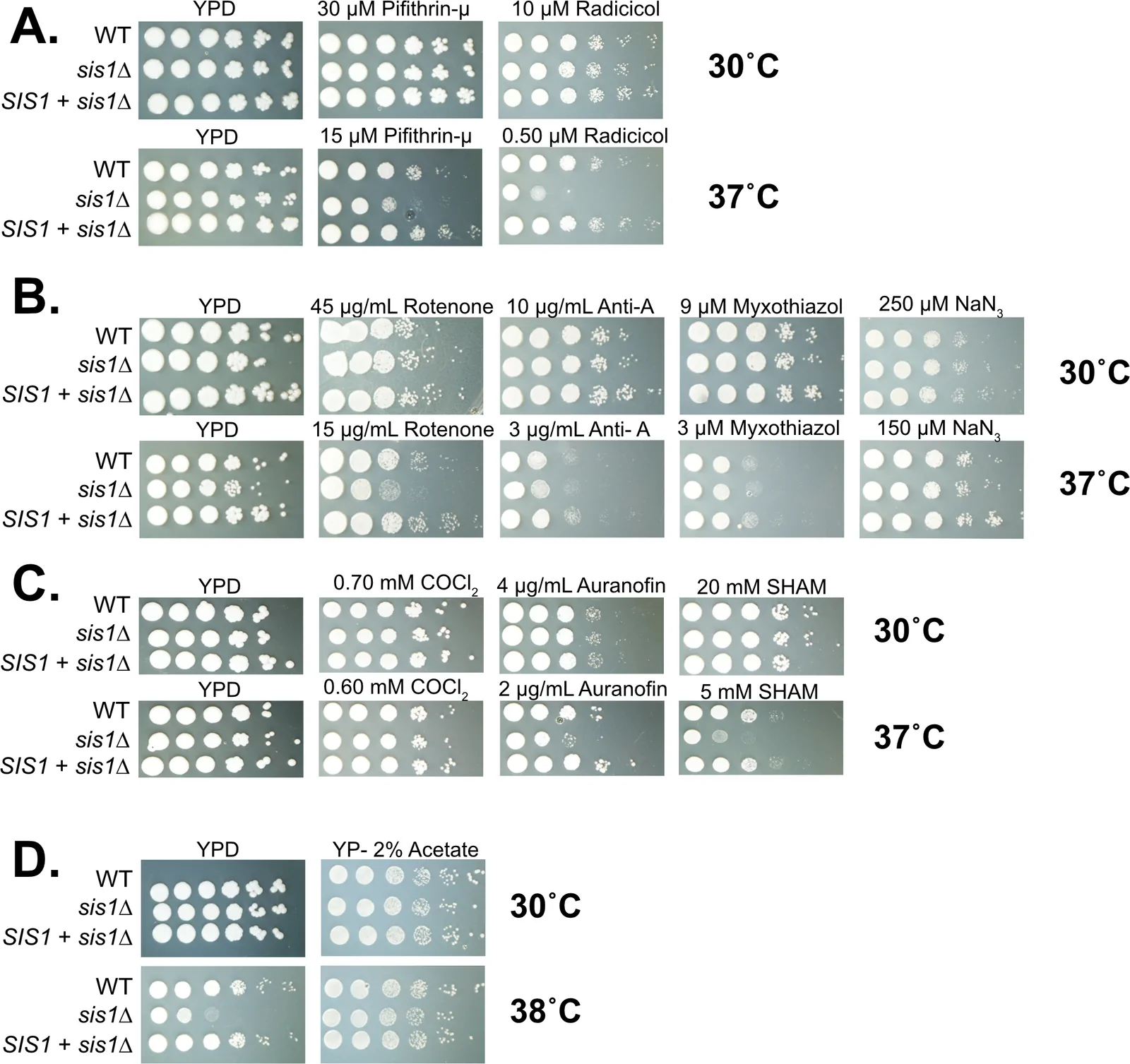
Hsp70/90 dysfunction and mitochondrial defects underlie the *sis1*Δ thermosensitivity. (**A**) Serial dilution spot plate analysis of WT, *sis1*∆, and *SIS + sis1*∆ on YPD agar grown at the indicated temperature in the presence of drugs affecting Hsp70s (Pifithrin µ) or Hsp90s (Radicicol). Shown is one representative biological replicate (*n* = 3). (**B and C**) Serial dilution spot plate analysis of WT, *sis1*∆, and *SIS + sis1*∆ on YPD agar grown at the indicated temperature in the presence of drugs affecting the mitochondrial electron transport chain or mitochondrial function. Shown is one representative replicate (*n* = 3). (**D**) Serial dilution spot plate analysis of WT, *sis1*∆, and *SIS + sis1*∆ on YP agar with 2% acetate as the sole carbon source. Shown is one representative biological replicate (*n* = 3).

To further investigate the impact of *SIS1* deletion on cellular processes that impact thermotolerance, we assessed the sensitivity of the *sis1*Δ mutant to cell integrity stress and mitochondrial stress (37–41). Spot dilution analysis of the wild-type, *sis1*Δ mutant, and complemented strains on media containing the cell wall stressors (6) calcofluor white, Congo red, SDS, and caffeine revealed no overt sensitivity or exacerbation at 37°C, suggesting that the thermosensitivity of the *sis1*Δ mutant is not due to cell integrity defects (File S6).

One of the defined cellular roles of Sis1 in the model yeast is to promote the unfolded state of nuclear-encoded mitochondrial proteins to facilitate import into the mitochondrion (42, 43). Sis1 interacts with newly synthesized mitochondrial beta-barrel proteins, with its role as an Hsp40 chaperone in yeast (43). Given this role, we next assessed whether the *sis1*Δ mutant exhibited sensitivity to mitochondrial stressors. We compared the growth of wild-type, *sis1*Δ, and the complemented strains on agar containing electron transport chain inhibitors, including Rotenone (Complex I), Myxothiazol (Complex III) (44), Antimycin A (Complex III) (45), and NaN_3_ (Complex IV) (46) (Fig. 5B). We found that the *sis1*Δ mutant exhibited sensitivity to Rotenone specifically at 37°C, with no sensitivity to the other complex inhibitors at 37°C. In addition, we compared growth in the presence of CoCl_2_ (hypoxia mimetic) (47), Auranofin (Mia40-Erv1 pathway and Pos5 NADH Kinase) (48, 49), and SHAM (alternate oxidase) (50) (Fig. 5C). We found that the *sis1*Δ mutant exhibited sensitivity to both SHAM and Auranofin specifically at 37°C. To investigate whether the thermosensitivity of the *sis1*Δ mutant was connected to the mitochondrial defects, we assessed the impact of acetate as the sole carbon source, which forces carbon flux through the TCA cycle (51), on the growth of the wild-type, *sis1*Δ mutant, and complemented strains at 30°C and 38°C. We found that growth on acetate as the sole carbon source suppressed the thermosensitivity of the *sis1*Δ mutant, suggesting that mitochondrial defects, either in addition to or resulting from Hsp70/90 dysfunction, underlie the thermosensitivity of the *sis1*Δ mutant (Fig. 5D).

## DISCUSSION

For *C. neoformans* to successfully survive the insults of the human host, it must rapidly reprogram its translatome with stress effector proteins (6, 11, 14). Our previous work informed a model of global translatome reprogramming as a highly regulated process involving the collaboration of Gcn2 and Ccr4 (11–13), regulating entry and exit of mRNAs into the translating pool, respectively. How specific stress-dependent ribosome interactors impact stress adaptation and pathogenesis in fungal pathogens such as *C. neoformans* at a more granular level remains unexplored. In this study, we sought to investigate the recruitment of stress-specific factors to the fungal ribosome in *C. neoformans* and explore their role in translatome reprogramming, stress adaptation, and pathogenesis using RiboPROT, a mass spectrometry-based approach. We identified proteins that associate with ribosomes uniquely during thermal and oxidative stress, as well as shared interactions, and found the heat shock protein chaperone Sis1 as a contributor to thermotolerance and virulence in *C. neoformans*.

There are many challenges associated with treating *C. neoformans* infections, including host toxicity and antifungal resistance, that highlight the urgent need for novel antifungal drugs and the identification of unique drug targets. Protein synthesis is an essential and highly controlled process with ribosomes at the center, but targeting translation in a eukaryotic organism poses a risk of host toxicity due to the evolutionary conservation of the core machinery (7). Finding unique differences between fungal and human protein synthesis would lead to drug targets for the development of novel therapeutics.

Ribosomes are now recognized as regulatory platforms (15, 52, 53), rather than passive protein synthesis machines, with the recruitment of specific factors during stress modulating ribosome function (16, 17). Applying this framework to a fungal pathogen, we developed and validated the RiboPROT approach to identify stress-specific ribosome-associated proteins in *C. neoformans*.

Initial screening of deletion mutants in select temperature-induced ribosome-associated factors revealed that a *sis1∆* mutant was thermosensitive at human febrile temperature. Thermotolerance is an essential virulence trait (29). As a heat shock protein, the role of Sis1 becomes increasingly important during thermal stress, as evidenced by induced *SIS1* expression following a shift to 37°C and attenuated virulence in the *G. mellonella* model at 38°C.

To mitigate proteotoxic stress in the cell, transcripts that encode Hsps are upregulated as part of the heat shock response. By acting in a network, Hsps act as molecular chaperones that promote protein folding, stabilization, and transport to maintain growth and preserve protein integrity during stress (54, 55). Sis1 is a J domain heat shock protein chaperone (18), a family of 40 kDa proteins that act as co-chaperones with Hsp70s and are characterized by their highly conserved ~70 amino acid “J,” bearing a histidine-proline-aspartic acid tripeptide motif that is imperative to their Hsp70 stimulatory activity (33). J domain proteins, such as Zuo1, act at the ribosome to assist in folding nascent polypeptides (56). Interestingly, *SIS1* is essential in *S. cerevisiae* (57), suggesting evolutionary divergence between species. The observed sensitivity of the *sis1*Δ mutant to inhibitors of Hsp70 and Hsp90 family members, coupled with sensitivity to mitochondrial perturbation, is consistent with a role for Sis1 in promoting the folding and/or mitochondrial import of select nuclear-encoded mitochondrial proteins, as has been reported in *S. cerevisiae* (43, 58, 59). Although we observed a higher magnitude of transient repression in translation initiation in the absence of Sis1 during thermal stress via polysome profiling, it appears to be Gcn2-independent, as there was no observed difference in eIF2α phosphorylation between wild type and the *sis1*Δ mutant at the same time point. This modest effect was absent at 38°C, again suggesting the role of Sis1 in regulating a specific subset of *C. neoformans* proteins rather than affecting global translation regulation.

The identification of Sis1 as a ribosome-associated protein under thermal stress, together with the resulting thermosensitivity and mitochondrial dysfunction, is consistent with previously identified roles for this J-domain heat shock co-chaperone (19, 20, 43, 58, 60).

A limitation of this study is that direct confirmation of Sis1 ribosomal association in *C. neoformans* could not be biochemically validated via RAPPL due to the failure of the tagged strains to complement. However, Sis1 has been shown to associate with ribosomes in *S. cerevisiae* (19) and hold important roles in translation (20, 58, 61, 62). We did not observe global translational defects in the absence of Sis1, which may suggest that Sis1 is modulating the translation, folding, and localization of a specific subset of *C. neoformans* proteins that confer thermotolerance and promote mitochondrial function.

Together, the finding that growth on acetate as the sole carbon source suppressed the thermosensitivity phenotype at 38°C, despite showing sensitivity to multiple mitochondrion-perturbing drugs, contributes to the evidence that suggests Sis1 is linked to mitochondrial homeostasis through the maintenance of specific mitochondrial proteins during thermal stress. Future work will identify specific targets of Sis1 co-chaperone activity that promote thermotolerance and virulence using both proteomic and ribosome-associated transcriptomic approaches.

## MATERIALS AND METHODS

### Strains

Laboratory strain of *C. neoformans* H99 was used as our wild-type background. Strains were constructed using biolistic transformation unless otherwise noted, with the primer sequences noted in File S7. Verification of all strains included phenotypic analysis, PCR sequencing, and northern blot confirmation. The *sis1*Δ mutant in a KN99 background was obtained from the Madhani knockout collection (2016 Plate), purchased from the Fungal Genetics Stock Center. The *sis1*Δ mutant construct was PCR amplified using primers Sis1LinearReverse(KO)-CCAGTACCGAATGCCAACGCC and Sis1LinearForward(KO)-CACTGGAACTTGGATTTGCGG and transformed into our H99 background via biolistic transformation. Nourseothricin (NAT)-resistant colonies were screened for gene replacement via PCR. The *sis1*∆:*SIS1* complement strain was constructed by cloning the *SIS1* locus into the pBluescript vector containing the G418 (NEO) resistance cassette using XbaI digestion. Electroporation was used to transform the vector into the *sis1*∆ mutant cells following 48 h of incubation in YNB-2% dextrose media at 30°C for capsular shedding (63). Geneticin was used to select resistant transformants, which were further confirmed by PCR.

Plasmid cloning for the Sis1-N and C-terminally FLAG-tagged strains was performed by GenScript (Piscataway, NJ, USA). Whole plasmids were transformed into our *sis1*Δ mutant via biolistic transformation. Geneticin was used to select for resistant transformants, and spot plate analysis was used to phenotypically screen for functional FLAG-tagged mutants.

The CNAG_05167 mutant was constructed as described previously (64). Briefly, the nourseothricin cassette was digested with MunI and BglII, ligated to flanked upstream and downstream sequences to CNAG_05167, and the full knockout construct was ligated into pBluescript via digestion using XbaI and XhoI.

The *hsp104*Δ mutant was created similarly, except that a G418 (NEO) resistance cassette was flanked by upstream and downstream *HSP104* sequences. Primers used to amplify NEO and the flanking sequences can be found in File S7. To create the hsp104 knockout construct, 700 bp of sequence upstream of HSP104 was amplified with 5′ XbaI and 3′ BglII ends, and 700 bp of sequence downstream of HSP104 was PCR amplified with 5′ MunI and 3′ XhoI ends. The plasmid bearing the GIS2 knockout construct (63) was digested with XbaI and BglII to remove the upstream GIS2 arm, and the remaining plasmid was ligated with the upstream HSP104 arm. After sequence verification of insertion of the upstream HSP104 arm, the plasmid was digested with MunI and XhoI to remove the downstream GIS2 arm, and the remaining digested plasmid was ligated with the downstream HSP104 arm. The resulting HSP104 knockout construct consisted of the pBluescript backbone with the HSP104 upstream and downstream arms flanking a G418 resistance cassette. The HSP104-FLAG construct was first attempted using primer extension overlap PCR. HSP104 with 1 kb upstream and 3′ FLAG tag was amplified with primers F-HSP104-1kbup-XbaI and R-HSP104 + FLAG. The HSP104 terminator with a 5′ FLAG tag was amplified with F-FLAGstop-HSP104term and R-HSP104term-BamHI. Phusion PCR was followed with F-HSP104-1kbup-XbaI and R-HSP104term-BamHI. The fused product was ligated into pBluescript + NAT. Sequencing of plasmids from transformed *Escherichia coli* confirmed insertion of HSP104 with an in-frame FLAG tag and stop codon, followed by only the sequence corresponding to the reverse terminator primer. The plasmid was digested with BamHI, and the terminator amplified with F-HSP104term-BamHI and R-HSP104term-BamHI was inserted, with orientation verified by sequencing. The construct was transformed into the *hsp104*Δ mutant via biolistics. FLAG expression was verified via western blot.

### Media and growth conditions

For our RiboPROT experimental culture, cells were first grown overnight in 5 mL of YPD (1% yeast extract, 2% peptone, and 2% dextrose) at 30°C and 250 rpm in snap-cap tubes (Falcon Corning Brand). For the RiboPROT time courses, YNB (BD Difco cat. no. 291920) supplemented with 2% dextrose was used to control for potential protein contaminants in nutrient-rich media. For all further experiments, all cultures were first grown overnight in 5 mL of YPD at 30°C and 250 rpm in snap-cap tubes (Falcon Corning Brand). For time courses, liquid experimental cultures were seeded with an optical density at 600 nm of 0.20–0.22 in YPD. Cultures were grown to mid-logarithmic phase at 30°C for ~5 h with shaking at 250 rpm in baffled flasks, reaching an optical density at 600 nm of 0.55–0.60. Cultures were incubated and treated with a temperature shift (pre-warmed YPD at 37°C or 38°C). Cells were harvested by pelleting for 2 min at 3,000 rcf, then flash-frozen with liquid nitrogen and stored at −80°C. For spot plates, cells were plated on YPD agar (YPD, 2% agar) with the indicated drugs, temperatures, and times noted in the figures.

### Polysome profiling

The frozen cell pellets were mechanically lysed with cold polysome lysis buffer (20 mM Tris-HCl [pH 8.0], 140 mM KCl, 5 mM MgCl_2_, 0.5 mg/mL heparin sodium sulfate, 0.5 mM dithiothreitol [DTT], 0.1 mg/mL cycloheximide [Thermo; cat. no. 357420050], and 1% Triton X-100). The 10%–40% sucrose gradients (Biocomp Gradient Maker) were prepared similarly to the lysis buffer, without the addition of Triton X-100. A final concentration of 2 U/µL of SUPERase RNase inhibitor (Thermo) was added to the lysis buffer to protect RNA and clarify profile peaks. RNA concentrations were measured using a NanoDrop One, and 150 µg of RNA was loaded onto a 10%–40% sucrose gradient. The gradients were centrifuged at 39,000 rpm for 2 h at 4°C in a SW41-Ti swinging bucket rotor. To visualize profiles, gradients were run through a Piston Gradient Fractionator (Biocomp), and absorbance of gradients was measured at 260 nm. Shown is one representative biological replicate (*n* = 3).

### RiboPROT

Following culturing, cell lysate was layered on top of sucrose gradients and subjected to ultracentrifugation to separate ribosomal subunits, monosomes, and polysomes. A polysome profile with distinct peaks to resolve ribosomes and their subunits was created by running the gradient through a UV spectrophotometer. Ribosome-containing fractions from polysome profiling gradients were pooled, and the proteins were ethanol precipitated and analyzed by quantitative proteomics analysis, an approach we termed RiboPROT. We also pooled the same gradient regions from samples that were treated with EDTA to release mRNAs and dissociate the ribosome subunits such that they sediment early in the gradient. Proteomic analysis of these fractions was used to control for proteins that co-sediment with ribosomes in the gradient but are not ribosome-associated. To achieve reliable and in-depth characterization of the *C. neoformans* riboproteome, we devised RiboPROT, a peptide-centric bottom-up proteomics approach. In this approach, proteins were first cleaved into short peptides by proteolytic digestion. A unique surfactant-aided precipitation/on-pellet digestion protocol was adopted (65, 66). Before tryptic digestion, proteins were first denatured with surfactants, reduced, and alkylated to dissociate disulfide bonds, and then precipitated by organic solvents. A surfactant-aided digestion protocol was utilized to dissociate disulfide bonds before tryptic digestion and then precipitated by organic solvents to clear the solution of non-protein matrix, such as sucrose, from the polysome gradient. This was to ensure depletion of non-protein matrix components that may interfere with proteolytic digestion. Derived peptides were then separated by nano reversed-phase liquid chromatography (LC) and detected by mass spectrometry (MS). We employed a trapping nano LC-MS system for peptide detection as described previously (67). There are three fundamental components of this novel approach to increase the robustness of our analysis for the detection of potential low-abundance proteins: (i) a large-volume trapping column to ensure a high loading amount, sample cleanup, and peak focusing; (ii) a 3-h nano LC gradient on a 65-cm long column to provide high-resolution and reproducible peptide separation; and (iii) an ultra-high-field Orbitrap MS allowing sensitive peptide detection with low mass errors. Peptide sequences were identified via mapping the acquired MS spectra to all theoretical MS spectra of *C. neoformans* peptides generated by *in silico* cleavage of the *C. neoformans* protein sequence database. Peptide and corresponding protein abundance were determined with high confidence using the MS1 chromatographic peak area.

### RAPPL

This method of ribosome isolation and purification of associated proteins involves a low-salt buffer lysis, clarification of the lysate via centrifugation, and exposure to magnetic poly-lysine beads. Following washes and then an elution using a buffer containing poly-D/L-glutamic acid, the final product can be used for downstream applications (68). This method exploits the negative charge of the phosphate group backbone of RNA and positively charged amino groups of poly-lysine. Ribosome isolation was performed following the protocol by Erath et al. (68). As described for other temperature shift time courses, 30°C mid-logarithmic phase cultures were pelleted, with 15 mL removed as a mid-log untreated control, and the rest of the culture was resuspended in prewarmed YPD and continued to grow for 1 h. At the time of harvest, cells were centrifuged and then washed with phosphate-buffered saline (PBS) containing cycloheximide (Thermo; cat no. 357420050), and pellets were resuspended in RAPPL lysis buffer (100 mM HEPES KOH solution [pH 7.5], 50 mM KCl, 10 mM MgOAc_2_, 1% Triton-X, 1 mM DTT, 100 µg/mL cycloheximide [Thermo; cat. no. 357420050], 10 U/mL HALT Protease Inhibitor [Thermo], 40 U/mL RNaseOUT, 4 U/mL DNase1, and 20 U/mL SuperaseIN) and transferred to microcentrifuge tubes containing acid-washed beads to proceed to lysis. Following lysis, 10 µg of protein lysate was saved to run as an input control on the western blot. Then, equal amounts and concentrations of lysate were mixed with 100 µL of poly-lysine magnetic beads and rotated at 4°C for 30 min. Polylysine Magnetic Beads (MCLAB; cat. no. PLYSMB-50) were washed three times using a magnet and 250 µL of RAPPL wash buffer (100 mM HEPES KOH solution [pH 7.5], 50 mM KCl, 10 mM MgOAc_2_, 1 mM DTT, 100 µg/mL cycloheximide [Thermo; cat. no. 357420050], 10 U/mL HALT Protease Inhibitor [Thermo], 40 U/mL RNaseOUT, and 20 U/mL SuperaseIN). Then, for elution, beads were resuspended in 30 µL of RAPPL elution buffer for 15 min on ice with gentle agitation (100 mM HEPES KOH solution [pH 7.5], 50 mM KCl, 10 mM MgOAc_2_, 1 mM DTT, 100 µg/mL cycloheximide [Thermo; cat. no. 357420050], 10 U/mL HALT Protease Inhibitor [Thermo], 40 U/mL RNaseOUT, 20 U/mL SuperaseIN, and 2 mg/mL poly-D-glutamic acid). Input and RAPPL samples were saved in 1× Laemmli buffer with 2-mercaptoethanol and run as previously described. Shown is one representative biological replicate (*n* = 3).

### Spot plate analysis

Overnight strains were grown in YPD overnight at 30°C. The next morning, cells were pelleted at 3,000 rcf for 2 min and washed twice with 5 mL of sterile deionized water. In 6 mL of sterile deionized water, the optical density at 600 nm of each culture was set to 1.0. The cultures were serially diluted 10-fold six times in sterile deionized water. Five microliters of the dilution was spotted on YPD agar plates. YP agar plates (YP, 2% agar) were supplemented with 2% acetate as an alternate carbon source. Drugs added to the plates are noted in figures, and all plates were incubated for 72 h at 30°C, 37°C, or 38°C. Shown is one representative biological replicate (*n* = 3).

### Northern blotting

For temperature shift time courses, 30°C mid-logarithmic phase cultures were pelleted, with 10 mL removed for time point 0; the rest was resuspended in prewarmed YPD, and 10 mL was placed in pre-warmed Falcon Snap Caps in the 37°C incubator at 250 rpm. At the indicated time points, 5 mL of cells was pelleted and flash-frozen. At the time of extraction, cells were thawed on ice, and RNA was harvested as previously described (6, 11). Three micrograms of RNA from each sample was loaded on an RNA gel, transferred to a nylon membrane overnight, crosslinked, then blots were probed against *RPL2*, *SSA2*, or *SIS1* ^32^P-labeled probes, as indicated in the figure. Probes were created using genomic DNA amplified using primers in File S7. Signal was detected via a phosphor screen and a Typhoon imager (GE) and quantified with ImageLab software 6.1 (Bio-Rad). rRNA was used as a loading control visual; shown is one representative biological replicate (*n* = 3).

### Growth curve analyses

Overnight cultures were diluted to an optical density at 600 nm of 0.1 in YPD, and 100 µL of each strain was placed into a 96-well plate (Falcon Corning Brand) in triplicate. Plates were incubated at either 30°C, 37°C, or 38°C with linear shaking in a BioTek Synergy Neo plate reader. An OD_600_ reading was recorded every 15 min for 24 h. Growth rate was calculated during the exponential growth phase (~8–13 h) across three biological replicates. Rate = ln(OD_600_[final]/OD_600_[initial])/duration, as previously described (13).

### *G. mellonella* survival assay

Overnight-grown cultures of *C. neoformans* strains (H99, *sis1∆*, and *sis1Δ*::SIS1) were washed twice with phosphate-buffered saline and resuspended in 1 mL of PBS. Cells were counted using a hemocytometer, and the inoculum was prepared by diluting the cells to 10^4^ cells/µL. For each strain, 10 *G. mellonella* larvae (190–250 mg) were infected by injecting 10^5^ cells (10 µL) into the last left proleg. The larvae were incubated at 30°C, 37°C, and 38°C for 7 days. For a control, 10 µL PBS was injected into the larvae subset. The experiment was performed in biological triplicate. Survival curves were plotted using GraphPad Prism 10 software. Differences in survival between groups were assessed using the log-rank (Mantel–Cox) test. *P* values < 0.05 were considered significant. Shown is one representative biological replicate (*n* = 3).

### Western blotting

For temperature shift time courses, 30°C mid-logarithmic phase cultures were pelleted, with 10 mL removed for time point 0; the rest was resuspended in prewarmed YPD, and 10 mL was placed in pre-warmed Falcon Snap Caps in the 37°C or 38°C incubator at 250 rpm, as specified in the figure. At the indicated time points, cells were pelleted at 3,000 rcf for 2 min, the supernatant was removed, then pellets were flash-frozen with liquid nitrogen and stored at −80°C. Pellets were thawed on ice, lysed, and subjected to SDS-PAGE as previously described (6). To assess phosphorylated eIF2α, a rabbit α-Phospho-eIF2α primary antibody (Abcam, cat. no. ab32157; 1:1,000 dilution) was used, and an Anti-rabbit IgG, HRP-linked secondary antibody (Cell Signaling Technologies, cat. no. 7074; 1:10,000 dilution). Membranes were stripped using Restore Western Blot Stripping Buffer (Thermo, cat. no. 21059) and re-probed for total eIF2α with a custom primary rabbit antibody (GenScript). HRP signal detection was measured using a Bio-Rad Chemidoc MP and Clarity Max Western ECL substrate (Bio-Rad, cat. no. 1705062). Shown is one representative biological replicate (*n* = 3).

## Data Availability

The mass spectrometry proteomics data have been deposited in the ProteomeXchange Consortium via the PRIDE partner repository with the data set identifier PXD082145.

## SUPPLEMENTAL MATERIAL

File S1 spectrum.00104-26-s0001.xlsx Proteomics data.

File S2 spectrum.00104-26-s0002.xlsx GO analysis.

File S3 spectrum.00104-26-s0003.xlsx Proteins that meet the criteria for hit calling.

File S4 spectrum.00104-26-s0004.tiff FLAG complementation of the sis1 Delta mutant.

File S5 spectrum.00104-26-s0005.tiff Mapping of peptides on selected hits.

File S6 spectrum.00104-26-s0006.tiff Cell wall panel of the sis1 mutant.

File S7 spectrum.00104-26-s0007.pdf Oligonucleotide sequences.

Supplemental legends spectrum.00104-26-s0008.docx Legends for Files S1 to S7, Tables S1 and S2.

Table S1 spectrum.00104-26-s0009.tiff Ribosome-associated GO.

Table S2 spectrum.00104-26-s0010.pdf Ribosome-associated proteins under thermal stress.

ASM does not own the copyrights to Supplemental Material that may be linked to, or accessed through, an article. The authors have granted ASM a non-exclusive, world-wide license to publish the Supplemental Material files. Please contact the corresponding author directly for reuse.
