# Supplementary material for: Stress-dependent ribosome-associated proteomics reveals a role for Sis1 in thermotolerance and virulence in Cryptococcus neoformans
Source: Microbiol Spectr. 2026 Sep 21;14(11):e00104-26. doi: 10.1128/spectrum.00104-26 (PMC13729215; doi:10.1128/spectrum.00104-26)
Supplement: Supplementary file 1 [file spectrum.00104-26-s0007.pdf]

Oligonucleotide Primer Sequences

Primer	Sequence 5' – 3'
SIS1 Northern Blot Forward	CTGACATCCGCAGACCCTGGGT
SIS1 Northern blot Reverse	ACCTCCTTAGTGATGGCACCGC
SSA2 5' UTR Northern Blot Forward	CGCAGCTTGTGGGACGT
SSA2 5' UTR Northern Blot Reverse	GGTGTGTGTATATGTGCTGTTGTGCT
RPL2 Forward	ATGGGTCGAGTCATCCGCGC
RPL2 Reverse	TTAAGCGTTGTCGACGTTCTTGC
Sis1 Linear Reverse (KO)	CCAGTACCGAATGCCAACGCC
Sis1 Linear Forward (KO)	CACTGGAACTTGGATTTGCGG
F-Sis1 1kb up (Xbal) (Complement)	TAATAATCTAGAGCAGCTGTTGATGACACGACC
R-Sis1 1kb down (Xbal) (Complement)	TAATAATCTAGAGGATTAATAGTGGTACAGGACGGC
05167_Up_Forward (Xbal) (KO)	TAATAATCTAGACGGTCGAACCCAAATAAACCTTGG
05167_Up_Reverse (BglII) (KO)	TAATAAAGATCTATTGGCTTCCATCTTGGGGTGACG
05167_Down_Forward (KO) (Mun1)	TAATAACAATTGCGTAACGGCGCAAGCTGAACATAG
05167_Down_Reverse (Xhol) (KO)	TAATAACTCGAGACCCAGAGCCTTGGGAGAGAAACC
F-HSP104700up-KO-XbaI	TAATAATCTAGACAGACTCTCACACCGAAATGC
R-HSP1047up-KO-BglII	TAATAAAGATCTATGGTTGGTGGGAGAGCG
F-HSP104down-KO-MunI	TAATAACAATTGAAGGCGGCAGGTCTGATCATCG

R-HSP104down-KO-XhoI	TAATAACTCGAGGTCCGGGCATTATGGGAGG
F-HSP104-1kbup-XbaI	TAATAATCTAGAGCCATTTCTCAGCATAACGC
R-HSP104+flag	CTTGTCATCGTCATCCTTGTAATCATCCATTTCTTCCACCTCAATATCC
F- FLAGstop-HSP104term	GATTACAAGGATGACGATGACAAGTAATGTGCCCAAGGCGGCAGG
R-HSP104term-BamHI	TAATAATCTAGAACCACCTACGGGATCATCTACCC
F-HSP104term-BamHI	TAATAAGGATCCTGTGCCCAAGGCGGCAGG
R-HSP104term-BamHI	TAATAAGGATCCACCACCTACGGGATCATCTACCC
