# Supplementary material for: Stress-dependent ribosome-associated proteomics reveals a role for Sis1 in thermotolerance and virulence in Cryptococcus neoformans
Source: Microbiol Spectr. 2026 Sep 21;14(11):e00104-26. doi: 10.1128/spectrum.00104-26 (PMC13729215; doi:10.1128/spectrum.00104-26)
Supplement: Supplementary file 2 [file spectrum.00104-26-s0010.pdf]

Table 2

Alternate ID	Identified Proteins: Temperature
CNAG_04032	AFG3 family protein, AFG3 LIKE MATRIX AAA PEPTIDASE SUBUNIT 2, ISOFORM A,metalloprotease#PC00153;protease#PC00190;protein modifying
CNAG_05167	Hypothetical Protein
CNAG_06106	chaperone regulator, DNAJ HOMOLOG SUBFAMILY B MEMBER 5, Sis 1
CNAG_05753	ARG5,N-acetyl-gamma-glutamyl-phosphate reductase, variant, N-acetyl-gamma-glutamyl-phosphate reductase
CNAG_06112	Carbamoyl-phosphate synthase arginine-specific large chain, CAD PROTEIN
CNAG_00747	Succinyl-CoA synthetase beta subunit
CNAG_02916	F-type H -transporting ATPase subunit F
CNAG_07347	HEAT SHOCK PROTEIN 104, Heat shock protein
CNAG_05631	NADH-ubiquinone oxidoreductase 49 kDa subunit, mitochondrial
CNAG_01820	PYK1,pyruvate kinase, variant, Pyruvate kinase
CNAG_02833	NADH dehydrogenase (ubiquinone) 1 beta subcomplex
