# Supplementary material for: Stress-dependent ribosome-associated proteomics reveals a role for Sis1 in thermotolerance and virulence in Cryptococcus neoformans
Source: Microbiol Spectr. 2026 Sep 21;14(11):e00104-26. doi: 10.1128/spectrum.00104-26 (PMC13729215; doi:10.1128/spectrum.00104-26)
Supplement: Supplementary file 3 [file spectrum.00104-26-s0008.docx]

**Supplemental Material 1: Proteomics Data.** CNAG identifier IDs are listed for the STD experimental conditions and chelated controls for both spectral counts and normalized spectral abundance factor.

**Supplemental Material 2: GO Analysis.** GO terms with <0.05 BH p-value are highlighted in red.

**Supplemental Material 3: Proteins that meet the criteria for hit calling**. Found in 3/4 or more of replicates in each listed condition and not found in the chelated control groups.

**Supplemental Material 4: FLAG complementation of the sis1 mutant.** Spot dilution plate analysis of indicated strains on YPD agar. Overnight strains were grown in YPD media at 30˚C, washed and resuspended in sterile water to an OD600 of 1.0. Ten-fold serial dilutions were spotted on YPD plates and incubated for three days at the indicated temperature to assess complementation of the Flag tagged strains. Plates were photographed following 72 hours of growth. Shown is one representative biological replicate.

**Supplemental Material 5: Mapping of peptides on selected hits.** Sequence coverage for each protein hit shows no N-terminal bias for our selected hits, excluding the potential for our hits being nascent polypeptides.

**Supplemental Material 6: Cell wall panel of the sis1 mutant.** Spot dilution plate analysis of indicated strains on YPD agar was conducted as previously described. Ten-fold serial dilutions were spotted on YPD plates with the indicated drugs and incubated for three days at the indicated temperatures. Plates were photographed following 72 hours of growth. Shown is one representative biological replicate (n=3).

**Supplemental Material 7: Oligonucleotide sequences.** Primer sequences used in the generation and validation of strains.

**Table S1: Ribosome-associated GO.** The following table shows the major biological processes (BP), molecular function (MF), and cellular component (CC) GO term hits that predominated in the experimental sample groups. P value, Benjamini correction, and FDR are included per term in the table.

**Table S2: Ribosome-associated proteins under thermal stress.** Table shows filtered hits for the temperature condition.
